# Mapping the Binding Site of a Cross-Reactive *Plasmodium falciparum* PfEMP1 Monoclonal Antibody Inhibitory of ICAM-1 Binding

**DOI:** 10.4049/jimmunol.1501404

**Published:** 2015-08-28

**Authors:** Frank Lennartz, Anja Bengtsson, Rebecca W. Olsen, Louise Joergensen, Alan Brown, Louise Remy, Petr Man, Eric Forest, Lea K. Barfod, Yvonne Adams, Matthew K. Higgins, Anja T. R. Jensen

**Affiliations:** * Department of Biochemistry, University of Oxford, Oxford OX1 3QU, United Kingdom;; † Department of Immunology and Microbiology, Centre for Medical Parasitology, Faculty of Health and Medical Sciences, University of Copenhagen, Copenhagen 1014, Denmark;; ‡ Department of Infectious Diseases, Copenhagen University Hospital (Rigshospitalet), Copenhagen 2100, Denmark;; § Department of Biochemistry, University of Cambridge, Cambridge CB2 1GA, United Kingdom;; ¶ Institut de Biologie Structurale, Grenoble F-38044, France;; ‖ Institute of Microbiology, Academy of Sciences of the Czech Republic, 117 20 Prague, Czech Republic; and; # Faculty of Science, Charles University in Prague, 116 36 Prague, Czech Republic

## Abstract

The virulence of *Plasmodium falciparum* is linked to the ability of infected erythrocytes (IE) to adhere to the vascular endothelium, mediated by *P. falciparum* erythrocyte membrane protein 1 (PfEMP1). In this article, we report the functional characterization of an mAb that recognizes a panel of PfEMP1s and inhibits ICAM-1 binding. The 24E9 mouse mAb was raised against PFD1235w DBLβ3_D4, a domain from the group A PfEMP1s associated with severe malaria. 24E9 recognizes native PfEMP1 expressed on the IE surface and shows cross-reactivity with and cross-inhibition of the ICAM-1 binding capacity of domain cassette 4 PfEMP1s. 24E9 Fab fragments bind DBLβ3_D4 with nanomolar affinity and inhibit ICAM-1 binding of domain cassette 4–expressing IE. The antigenic regions targeted by 24E9 Fab were identified by hydrogen/deuterium exchange mass spectrometry and revealed three discrete peptides that are solvent protected in the complex. When mapped onto a homology model of DBLβ3_D4, these cluster to a defined, surface-exposed region on the convex surface of DBLβ3_D4. Mutagenesis confirmed that the site most strongly protected is necessary for 24E9 binding, which is consistent with a low-resolution structure of the DBLβ3_D4::24E9 Fab complex derived from small-angle x-ray scattering. The convex surface of DBLβ3_D4 has previously been shown to contain the ICAM-1 binding site of DBLβ domains, suggesting that the mAb acts by occluding the ICAM-1 binding surface. Conserved epitopes, such as those targeted by 24E9, are promising candidates for the inclusion in a vaccine interfering with ICAM-1–specific adhesion of group A PfEMP1 expressed by *P. falciparum* IE during severe malaria.

## Introduction

Human malaria caused by *Plasmodium falciparum* parasites remains a serious health problem. In 2013, an estimated 198 million cases of malaria resulted in 584,000 deaths, mostly in sub-Saharan Africa (1). The majority of deaths occurred in children <5 y of age.

Parasite virulence is linked to the ability of infected erythrocytes (IE) to adhere to the inside of host blood vessels, leading to inflammation, tissue obstruction, and organ dysfunction (2). IE adhesion is mediated by the surface expression of *P. falciparum* erythrocyte membrane protein 1 (PfEMP1) proteins, which are able to bind to various host receptors present on the endothelium.

The multidomain PfEMP1 proteins are encoded by ∼60 divergent *var* genes and consist of Duffy-binding–like (DBL) and cysteine-rich interdomain region protein domains (3), which can be divided into several major types (α, β, γ, etc.) and subtypes based on sequence similarities (4, 5). DBL domains generally contain three subdomains, which fold together to form a conserved α-helical core with loop insertions of variable sequence and length. Specific DBL and cysteine-rich interdomain region domains group together to form domain cassette (DC) families that are found across parasite isolates (5).

A frequently described PfEMP1 receptor is ICAM-1, and binding of IE to ICAM-1 during infection is linked to the development of symptoms of severe malaria, such as cerebral malaria (6–8). ICAM-1 is a membrane-bound protein with five extracellular domains (D1-D5) and is expressed by endothelial cells and leukocytes. ICAM-1 mediates leukocyte adhesion and migration to inflamed sites by binding to LFA-1 and Mac-1 (9, 10).

Surface expression of the recently identified DC4 containing PfEMP1s leads to ICAM-1–specific adhesion of IE, which is mediated by the DBLβ3_D4 PfEMP1 domain (11, 12) and appears to be involved in the pathogenesis of severe disease (13). Naturally acquired Abs against DC4 DBLβ3_D4 are cross-reactive and cross-inhibitory of ICAM-1 binding across members of DC4 and other DC types (12), suggesting that the DC4 DBLβ3 domains are attractive vaccine candidates.

Although no crystal structure exists currently for a DBLβ::ICAM-1 complex, this interaction has been studied in a number of different ways. Studies with truncated or mutated ICAM-1 constructs show that the binding site for DBLβ domains locates to the D1 domain of ICAM-1, and experiments with truncated and chimeric proteins have mapped the ICAM-1 binding site to the C-terminal end of DBLβ (14–17). In addition, ICAM-1 binding is gained when replacing the C-terminal subdomain of an ICAM-1 nonbinding DBLβ3 with that of the ICAM-1 binding PFD1235w DBLβ3_D4 (12). Homology modeling (18) and small-angle x-ray scattering (SAXS) (19), together with mutagenesis studies (20), further suggest that the interaction surface is on the convex surface of the DBLβ domain. However, the exact amino acids involved in DBLβ binding to ICAM-1 are yet to be determined.

The identification of DBLβ region(s) targeted by protective Abs and a detailed mapping of ICAM-1 binding epitopes will be an essential step toward designing a PfEMP1-based vaccine potentially protective against malaria. Using modern methods for the characterization of Ab–Ag complexes, such as hydrogen/deuterium exchange mass spectrometry (HDX MS), surface plasmon resonance (SPR), and SAXS, we characterized an mAb (24E9) that binds to the convex surface of DC4 DBLβ3_D4 domains and interferes with the DBLβ::ICAM-1 interaction. We show that 24E9 mAb targets epitopes conserved between DC4 DBLβ domains from genetically distant parasite isolates and inhibits ICAM-1 binding of IE by blocking the predicted ICAM-1 binding site on DBLβ. This provides important knowledge for choosing components for a vaccine aimed at preventing PfEMP1-mediated adhesion of IE during severe malaria.

## Materials and Methods

### Recombinant protein expression and purification

Full-length, wild-type PFD1235w DBLβ3_D4 was subcloned into a modified pET15b vector and expressed as an N-terminal, hexahistidine-tagged protein in *Escherichia coli* SHuffle 3030 cells (New England Biolabs) for 16 h at 25°C. The cells were pelleted, washed, and lysed, and DBLβ3_D4 was purified using Ni-NTA-Sepharose (QIAGEN). The hexahistidine-tag was removed by overnight cleavage at 4°C using Tobacco etch virus protease. Tobacco etch virus protease and uncleaved protein were removed by reverse immobilized-metal affinity chromatography, and DBLβ3_D4 was further purified by size exclusion chromatography using a Superdex 75 16/60 column (GE Healthcare).

PFD1235w DBLβ3_D4 protein used for mouse immunization to generate hybridomas was subjected to an additional purification step. DBLβ3_D4 was allowed to bind to ICAM-1_D1-D5-Fc coupled to a HiTrap NHS-activated HP column (GE Healthcare). Bound DBLβ3_D4 was eluted from ICAM-1 on the column and buffer exchanged into PBS.

For generation of DBLβ3_D4 mutants, a set of 5′ phosphorylated primers that included the coding sequence for the P2b or P3a regions of DBLβ3_D5 was used to amplify the DBLβ3_D4-encoding pEt15b vector by PCR. The PCR products were circularized by blunt-end ligation using T4 ligase (Life Technologies), and the mutants were expressed and purified as described for DBLβ3_D4.

ICAM-1 domains 1–5 (D1–D5) combined with the Fc region of human IgG1 (ICAM-1-Fc) was cloned, expressed, and purified as described previously (21). ICAM-1_D1-D2 was expressed in COS-7 cells and purified as described previously (19). ICAM-1_D1 was expressed and purified from *E. coli* BL21(DE3) as described previously (22).

### CD spectroscopy

Far-UV CD spectroscopy experiments were carried out with a J-815 Spectropolarimeter (Jasco) equipped with a computer-controlled Peltier temperature control unit. All samples were dialyzed into 10 mM sodium phosphate buffer, 150 mM NaF, pH 7.2, and measurements were taken at a protein concentration of 0.1 mg/ml using a 1 mM path cell. Spectra were acquired at 20°C at wavelengths between 195 and 260 nm. For thermal unfolding, the temperature was raised from 20° to 95°C in 0.5°C increments, and spectra were recorded between 200 and 250 nm wavelength.

### Hybridoma production

24E9 hybridomas were produced according to standard protocols (23). One CB6FI mouse (Harlan) was immunized s.c. with 30 μg PFD1235w DBLβ3_D4 in CFA (Sigma-Aldrich) followed by two additional boosters of 15 μg protein in IFA (Sigma-Aldrich). A final i.v. boost of 15 μg protein in PBS was given 3 d before the mouse was sacrificed and the spleen was taken out. Single spleen B lymphocytes were made from the whole spleen and fused to SP2/0-AG14 Myeloma cells (ATCC) in a 1:2 ratio using polyethylene glycol 4000. Spleen and myeloma cell mixture was diluted in 80 ml cell media (RPMI, 20% FBS, glutamine, penicillin/streptomycin) containing HAT media supplement (Sigma-Aldrich) to select for fused cells. A total of 100 μl/well was added to eight deep, flat-bottom, 96-well plates (Fisher Scientific) containing peritoneal macrophages from two BALB/C mice (Taconic) serving as feeder cells. Cells were grown for 1 wk at 37°C, 5% CO_2_ before changing the cell supernatant to cell media supplemented with HT media supplement (hypoxanthine and thymidine; Sigma-Aldrich). Two weeks after fusion, wells with growing cells were identified under a microscope and the cells were moved into fresh 96-well plates. After 1 wk, undiluted cell supernatant from each well was tested for the presence of DBLβ3_D4-reactive Abs using ELISA. To obtain true monoclonal hybridomas, we cloned cells from positive wells by limiting dilution. All animal procedures were approved by the Danish National Committee (Dyreforsøgstilsynet) in agreement with permit no. 2008/561-1498.

### mAb purification

24E9 monoclonal hybridomas were expanded and seeded at ∼10% confluency in 175-cm^2^ cell flasks containing 70 ml cell media [RPMI, 10% low IgG FBS (Lonza), HT media supplement (Sigma-Aldrich), glutamine, penicillin/streptomycin]. After incubation for 1 wk at 37°C, 5% CO_2_ cell supernatant was centrifuged, sterile-filtered, and buffer-exchanged into PBS before purifying mAb using a HiTrap protein G column (GE Healthcare) according to the manufacturer’s instructions.

### IgG subtyping

The IgG subtype and L chain class of 24E9 mAb were determined using an IsoQuick Kit for Mouse Monoclonal Isotyping (Sigma) according to the manufacturer’s instructions.

### Fab fragmentation

Purified 24E9 mAb was buffer-exchanged into cleavage buffer (0.1 M sodium phosphate pH 6.4, 0.3 M NaCl, 2 mM EDTA, 5 mM l-cysteine, 1.5 mM 2-ME) and concentrated to 1 mg/ml. Papain-agarose (Sigma-Aldrich) was added in a 20:1 ratio and incubated overnight at 37°C. Papain-agarose was removed by centrifugation, and the Fc portion and uncleaved mAb were removed from the supernatant by purification on a protein A column (GE Healthcare). Fab fragments in the flow-through were further purified by size exclusion chromatography.

### Western blot

Purified 24E9 mAb was tested for reactivity against reduced (+DTT) and nonreduced (−DTT) PFD1235w DBLβ3_D4. Purified PFD1235w DBLβ3_D4 and PFD1235w DBLβ3_D5 (control; 0.5 μg) were separated by SDS-PAGE under both conditions on a NuPAGE Novex 4–12% Bis-Tris gel in MOPS SDS Running buffer (Invitrogen) and subsequently blotted onto a Hybond-C Extra NC membrane (GE Healthcare). The membrane was blocked using 2.5% skimmed milk in dilution buffer (PBS, 1% BSA). The 24E9 mAb was diluted to 10 μg/ml in dilution buffer and added to the membrane. Bound 24E9 mAb was detected by anti-mouse IgG (P260; Dako) 1:1000 in dilution buffer using a chemiluminescent detection kit (Thermo Scientific).

### ELISA

#### Hybridoma screening.

Hybridoma cell supernatants were screened for PFD1235w DBLβ3_D4-reactive Abs using ELISA. Duplicate wells of MaxiSorp microtiter plates (Nunc) were coated with DBLβ3_D4 (50 μl; 1 μg/ml; 0.1 M glycine/HCl buffer pH 2.75; overnight; 4°C) and blocked with blocking buffer (PBS, 0.5 M NaCl, 1% Triton X-100, 1% BSA, pH 7.2). A total of 100 μl undiluted cell supernatant was added (1 h; room temperature). The plates were washed in PBS + 1% Triton X-100, and bound Ab was detected with an anti-mouse Ig-HRP (Dako; 1:3000 in blocking buffer). After 1 h of incubation, plates were developed using OPD tablets (Dako) according to the manufacturer’s instructions. The OD value was read at 490 nm using a VERSAmax microplate reader (Molecular Devices) and Softmax Pro v4.7.1.

#### mAb reactivity.

Microtiter plates were coated with 50 μl, 2 μg/ml recombinant proteins in glycine/HCl buffer and blocked with blocking buffer. 24E9 mAb (50 μl; 3-fold dilutions starting at 10 μg/ml; 1 h; room temperature) was added, and washing was performed as described above. Bound Ab was detected with anti-mouse Ig-HRP (Dako; 1:3000 in blocking buffer; 1 h; room temperature).

#### Reducing ELISA.

Microtiter plates were coated (50 μl; 2-fold dilutions starting at 64 μg/ml; glycine/HCl buffer; overnight; 4°C) with 24E9 mAb or the PFD1235w DBLγ-specific AB01 mAb (24) and blocked with PBS + 1% BSA. Plates were washed in PBS and PFD1235w DBLβ3_D4 or PFD1235w DBLγ (50 μl; 2 μg/ml; PBS ± 50 mM DTT; 1 h; room temperature) were added to the plates coated with 24E9 or AB01, respectively. Bound DBLβ3_D4 or DBLγ was detected by use of an anti–penta-His HRP Ab (1:3000 in PBS + 1% BSA; 1 h; room temperature; QIAGEN). Washing and detection were performed as described above.

#### ICAM-1 inhibition ELISA.

Microtiter plates were coated with recombinant ICAM-1–Fc (50 μl, 2 μg/ml; glycine/HCl buffer; overnight; 4°C) and blocked with blocking buffer. DBLβ3_D4 domains (1–16 μg/ml) were added simultaneously with mAb 24E9 added in 2-fold dilutions ranging from 0.25 to 32 μg/ml. Mouse IgG (Life Technologies) was added as control. ICAM-1–bound DBLβ3_D4 was detected using anti–penta-His HRP Ab (1:3000 in blocking buffer; 1 h; room temperature; QIAGEN). Washing and detection were performed as described above.

### Sequencing

The mouse Ig L and H chain variable genes of the 24E9 mAb were sequenced to determine the amino acid sequences of the CDRs. cDNA was made from single 24E9 hybridoma cells using a QIAGEN OneStep RT-PCR Kit with degenerate primers designed to target mouse Ig variable regions (25). cDNA was amplified using Phusion HF polymerase (New England Biolabs), and PCR products were sequenced using BigDye Terminator v3.1 Cycle Sequencing Kit (Applied Biosystems) according to the manufacturer’s instructions. Sequence data were collected on a 3100-Avant Genetic Analyzer (Applied Biosystems). The nucleotide sequences of 24E9 CDRs can be retrieved from GenBank using accession numbers KJ418726 (H chain) and KJ418727 (L chain) (http://www.ncbi.nlm.nih.gov/genbank).

### Malaria parasites and flow-cytometry analysis

The 3D7 *P. falciparum* clone and one Ghanaian patient isolate (BM057) were cultured in vitro (26) and were selected for DC4 PfEMP1 IE surface expression by repeated Ab selection as described previously (12). The identity of the isolates was routinely verified by genotyping as described previously (27), and *Mycoplasma* infection was regularly excluded using the MycoAlert *Mycoplasma* Detection Kit (Lonza) according to the manufacturer’s instructions.

*P. falciparum* IE were DNA-labeled with ethidium bromide and surface-labeled with mouse antisera obtained from the immunized mouse used for hybridoma production (15 μl serum/well), 24E9 mAb (100 μg/ml), or 24E9 Fab fragments (100 μg/ml). Whole Abs were labeled using an FITC-conjugated secondary anti-mouse IgG (1:100; Vector Labs), and an anti-mouse F(ab′)_2_ IgG (1:100; Jackson Immunoresearch) was used to detect Fab fragments. FITC fluorescence data from ethidium bromide^+^ cells were collected on a Cytomics FC 500 MPL flow cytometer (Beckman Coulter) and analyzed in WinList version 6.0 (Verity Software House).

### ICAM-1 adhesion assays under physiological flow conditions

Biochips (Vena8; Cellix) were coated at 4°C overnight with recombinant ICAM-1–Fc (50 μg/ml) produced as described previously (21). Channels [400 × 100 × 20 mm (w × d × l)] were blocked for 1 h at 37°C with PBS + 1% BSA and the chip mounted onto a Leica inverted phase-contrast microscope. To generate a wall shear stress representing that within microvasculature (1 dyn/cm^2^), we connected the biochip to an NE-1002X microfluidic pump (World Precision Instruments, U.K.). Erythrocytes at 3–5% parasitemia (1% hematocrit in RPMI 1640 plus 2% normal human serum) were flowed over the biochip for 5 min. The number of bound IE per square millimeter for five separate fields was counted at 20 times magnification, and a minimum of three independent experiments was done in triplicates.

To inhibit ICAM-1 adhesion, we combined IE with 24E9 mAb (1 × 10^−1^; 1, 10 μg/ml) or Fab fragments of 24E9 (1 × 10^−3^; 1 × 10^−1^; 1 μg/ml) before assaying as described earlier. Mouse IgG (10 μg/ml; Life Technologies) or mouse IgG Fab fragments (1 μg/ml; Rockland) were included as negative controls. Specificity of adhesion to recombinant ICAM-1–Fc was determined by the preincubation of channels with 40 μg/ml anti–ICAM-1 (clone 15.2; AbD Serotec).

### SPR

SPR measurements were conducted using a BIAcore T-100 instrument (GE Healthcare). DBLβ3_D4 was diluted into 10 mM acetate buffer, pH 4.0, and covalently coupled to a CM5 chip (GE Healthcare) by amine coupling to a density of 400 response units (RU). ICAM-1_D1, ICAM-1_D1-D2, and mAb 24E9 Fab were prepared in 10 mM HEPES pH 7.2, 150 mM NaCl, 50 μM EDTA, and 0.05% Tween 20. For each protein, a concentration series (100, 50, 25, 12.5, and 6.25 μg/ml) was flowed over the chip surface at a flow rate of 45 μl/min with an association time of 120 s and a dissociation time of 400 s. The signal from an empty flow cell was subtracted from all measurements. Between runs, the sensor surface was regenerated with 4 M MgCl_2_ for 30 s at a flow rate of 30 μl/min for the 24E9 Fab::DBLβ3_D4 interaction, or 5 mM NaOH for 10 s at a flow rate of 30 μl/min for the ICAM-1_D1::DBLβ3_D4 and ICAM-1_D1-D2::DBLβ3_D4 interactions.

For analysis of DBLβ3_D4 mutant binding to 24E9 mAb, 24E9 mAb was immobilized to 230 RU on a CM5 chip (GE Healthcare) precoupled with protein G. DBLβ3_D4 mutants were diluted into 10 mM HEPES pH 7.2, 300 mM NaCl, and 0.05% Tween 20, and for each mutant a concentration series (31.25, 15.62, 7.81, 3.90, 1.95, and 0.97 nM) was flowed over the chip surface at 40 μl/min with 240 s association time and 400 s dissociation time. The signal from a flow cell lacking the DBLβ3_D4 domain was subtracted from all measurements. The sensor surface was regenerated between runs with 100 mM glycine-HCl, pH 2.0 for 120 s at a flow rate of 10 μl/min.

For all measurements, sensorgrams corresponding to at least four different concentrations were globally fitted into a one-site kinetic model, and the values for *k*_a_, *k*_d_, and *K*_D_ were obtained using the BIAevaluation software 2.0.3 (GE Healthcare).

### HDX MS

HDX MS experiments were fully automated using a PAL autosampler (CTC Analytics). This controlled the start of exchange and quench reactions, proteolysis temperature (4°C), injection of the deuterated peptides, management of the injection and washing valves, and triggering of HPLC pumps and acquisition by the mass spectrometer. A Peltier-cooled box (4°C) contained two Rheodyne automated valves (6-port for injection and 10-port for washing), a desalting cartridge (peptide Opti-Trap Micro from Optimize Technologies), and an HPLC column (C18 Jupiter 4 μm Proteo 90 Å, 50 × 1 mm from Phenomenex). HDX MS reactions were carried out using gel-filtered DBLβ3_D4 and DBLβ3_D4::24E9 Fab (1:1 molar ratio), both at concentrations of 40 μM. Deuteration was initiated by a 5-fold dilution of DBLβ3_D4 or DBLβ3_D4::24E9 Fab (10 μl) with PBS in D_2_O (40 μl). The proteins were deuterated for 20 min at 4°C or 20 min at room temperature (26°C). Considering the change of exchange kinetics of amide hydrogens with temperature (about a 3-fold exchange increase for each 10°C increase in temperature), the last condition is equivalent to a 200-min deuteration at 4°C. A total of 50 μl 0.8 M Tris(2-carboxyethyl)phosphine, 2 M glycine was added for 10 min at 4°C to quench back-exchange and to reduce disulphide bridges. The proteins were digested online with immobilized porcine pepsin (Sigma) and recombinant nepenthesin-1 (28) proteases. The peptides were desalted using an HPLC pump (Agilent Technologies) with 0.03% trifluoroacetic acid in water (buffer A) at a flow rate of 100 μl/min. The peptides were then separated using another HPLC pump (Agilent Technologies) at 50 μl/min for 6 min with a 15–50% gradient of buffer B (buffer B: acetonitrile 90%, trifluoroacetic acid 0.03% in water), followed by 9 min at 50% B and 5 min at 100% B. The peptide masses were measured using an electrospray-time of flight mass spectrometer (Agilent 6210) in the 300–1300 m/z range. The peptides were previously identified by tandem mass spectrometry, using a Bruker APEX-Q FTMS (9.4 T). The Mass Hunter (Agilent Technologies) and Data Analysis (Bruker) software were used for data acquisition. The HD Examiner software (Sierra Analytics) was used for HDX MS data processing. For each deuteration time (20 min at 4°C or 20 min at room temperature), experiments were performed in triplicate and measurements were averaged.

### SAXS

SAXS measurements were carried out at the EMBL BioSAXS P12 beamline at the DORIS storage ring, DESY (Hamburg, Germany). Scattering data were recorded at a wavelength of 1.24 Å using a two-dimensional photon counting PILATUS 2 million pixel x-ray detector (Dectris, Baden, Switzerland). The distance between detector and sample was 3.1 m, resulting in a q range of 0.01–0.44 Å^−1^ [q = 4πsin(θ)λ^− 1^, where q is the scattering vector, 2θ is the scattering angle, and λ is the wavelength].

Samples for SAXS were prepared in buffer containing 20 mM HEPES, 150 mM NaCl, pH 7.4 and purified by size exclusion chromatography. Sample purity was verified by SDS-PAGE, and only samples with a purity >95% were used for data collection. Before measurement, samples were centrifuged at 13,000 rpm for 5 min at 4°C. For each sample, a concentration series (3.78, 1.79, 0.87, 0.34, 0.18 mg/ml for DBLβ3_D4 and 4.64, 2.38, 1.16, 0.59, 0.19 mg/ml for DBLβ3_D4::24E9 Fab) was measured at 10°C. Before and after each sample, buffer was measured as a control.

The scattering curves were manually inspected using PRIMUS (29), and frames showing signs of radiation damage were omitted in data analysis. Unaffected frames were averaged for each measurement, and the buffer signal was subtracted from the sample signal. To eliminate the effects of potential concentration-dependent protein aggregation at low scattering angles, the scattering curves of each concentration series were extrapolated to zero concentration. A composite curve was generated by scaling and merging the zero concentration curve with data for the highest concentration. The radius of gyration (Rg) was estimated by Guinier analysis using AutoRg in PRIMUS, whereas the maximum particle diameter (*D*_max_) and the pair distance distribution functions P_r_ were calculated using GNOM (30).

Ab initio models were generated from solution scattering data by DAMMIF (31) using default parameters with P1 symmetry. For both DBLβ3_D4 and DBLβ3_D4::24E9 Fab, 20 independent DAMMIF models were averaged using DAMAVER (32). The averaged model was further refined by DAMMIN (33), using default parameters and the original pair distance distribution functions as input. SITUS was used to calculate volumetric representation from the bead models generated by DAMMIN, and homology models of DBLβ3_D4 and a mouse Fab fragment (Protein Data Bank [PDB] ID 3GK8) were docked into the resulting envelopes using the program SCULPTOR (34). The DBLβ3_D4 homology model was generated with I-TASSER (c-score −0.9) using the structures of DBL3X (PDB ID 3BQK), NTS-DBL1α (PDB ID 2XU0), EBA-175 (PDB ID 1ZRL), and EBA-140 (PDB ID 4GF2) as templates. Structural models were visualized using PyMol Version 1.5.0.4 (Schrödinger).

## Results

### The 24E9 mAb is cross-reactive against DC4-containing PfEMP1 present on the surface of IE

**FIGURE 1.**
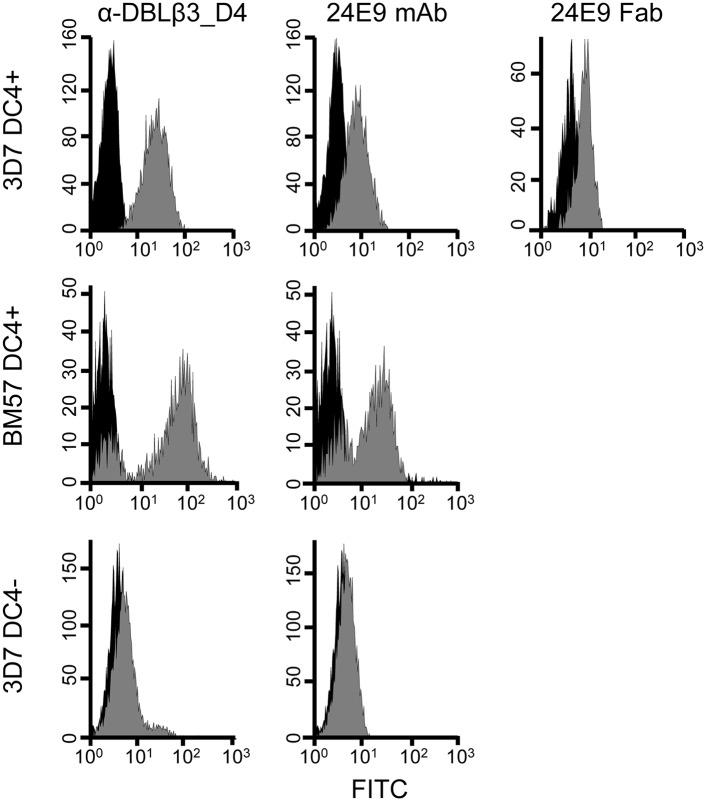
DC4-expressing *P. falciparum* IE are recognized by 24E9 mAb and Fab. PFD1235w DBLβ3_D4 mouse anti-sera and 24E9 mAb were tested by flow cytometry on 3D7 DC4^+^, BM57 DC4^+^, and 3D7 DC4^−^ parasite lines. 24E9 Fab was tested only on DC4^+^ 3D7. IE with antisera, 24E9 mAb, or 24E9 Fab are shown in gray, whereas IE without Abs (negative controls) are shown in black.

We have previously observed that 3D7 PFD1235w DBLβ3_D4 elicits adhesion-inhibitory Abs that are cross-reactive to DC4-containing PfEMP1 from genetically distant parasite isolates (12). To study the specific epitopes targeted by such protective Abs in more detail, we first raised a monoclonal mouse Ab against 3D7 PFD1235w DBLβ3_D4. This mAb, named 24E9, is of IgG1 isotype with κ L chains (data not shown). We tested the reactivity of 24E9 against native PfEMP1 on the surface of IE by flow cytometry. Both 24E9 mAb and Fab fragments generated from this Ab bound to erythrocytes infected with 3D7 parasites expressing DC4-containing PfEMP1 (Fig. 1). In contrast, 24E9 mAb did not recognize DC4^−^ 3D7 IE.

**FIGURE 2.**
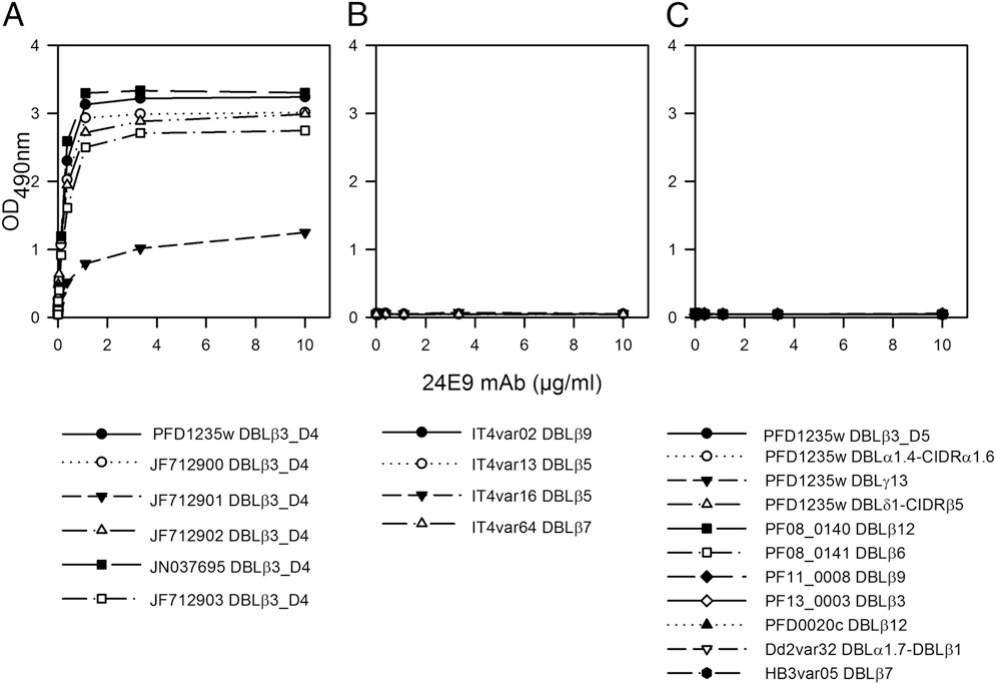
24E9 mAb is cross-reactive against DC4 DBLβ3_D4 domains. 24E9 mAb was tested against DC4-DBLβ3_D4 domains (**A**), non-DC4 DBLβ domains from the IT4 isolate (**B**) and non-DC4 domains from 3D7, Dd2, and HB3 isolates (**C**) using ELISA. Mean OD values are shown for three independent experiments. Error bars indicate SD.

24E9 mAb showed cross-reactivity to erythrocytes infected with the heterologous parasite strain BM057 (Fig. 1), which expresses a DC4 containing PfEMP1. We therefore used ELISA to test whether 24E9 mAb binds to other DBLβ domains. 24E9 mAb cross-reacted with five DBLβ3_D4 domains from DC4 containing PfEMP1 proteins cloned and expressed from Ghanaian field isolates (12) (Fig. 2A), but not with non-DC4 DBLβ domains from the IT4 isolate (Fig. 2B) or other PfEMP1 domains from the 3D7, Dd2, or HB3 isolates (Fig. 2C). Therefore, 24E9 mAb can recognize native PfEMP1 expressed on the surface of IE and shows patterns of cross-reactivity to DC4-containing PfEMP1s similar to Abs found in pooled immune plasma from patients infected with parasites expressing 3D7 PFD1235w (12).

### The interaction between DC4 DBLβ3_D4 and ICAM-1 is inhibited by 24E9

**FIGURE 3.**
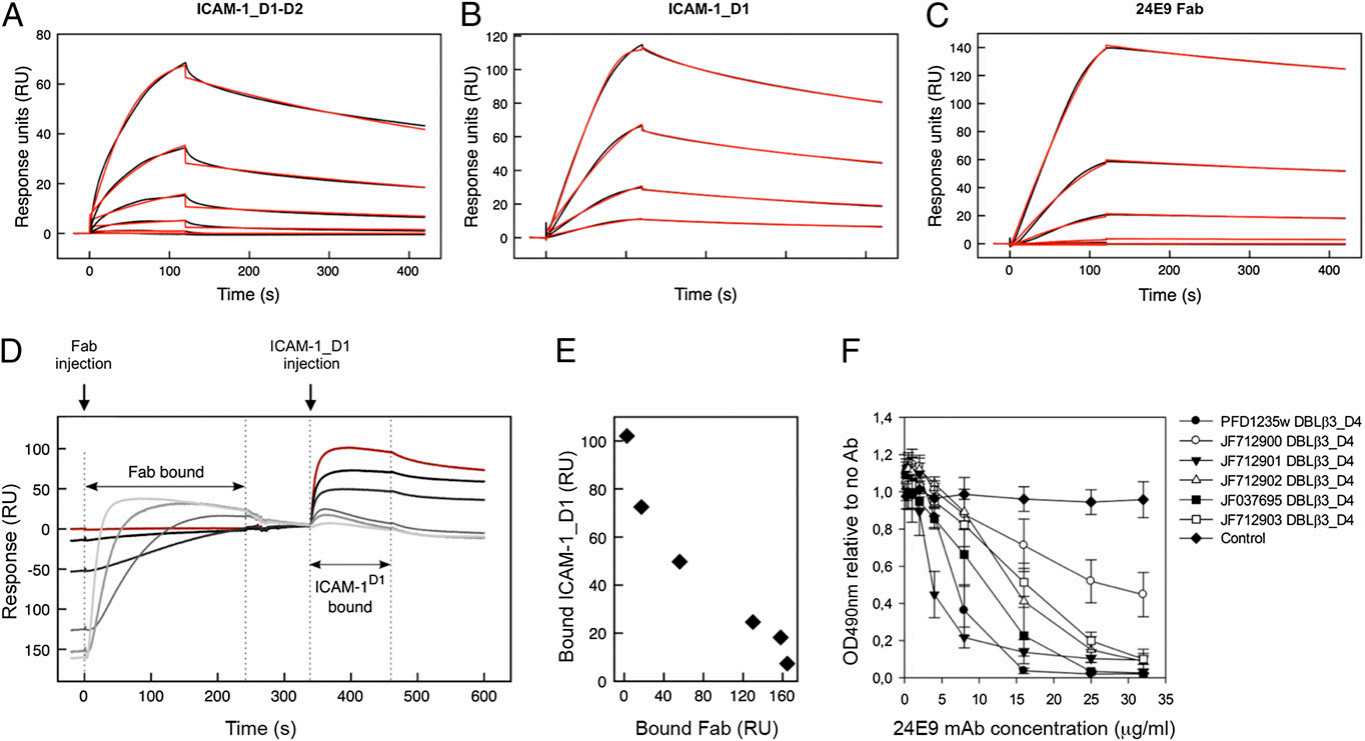
24E9 mAb inhibits ICAM-1 binding of DC4 DBLβ3_D4. PFD1235w DBLβ3_D4 was coupled to a sensor chip surface (RU = 400). Analytes were injected at 45 μl/min with an association phase of 120 s and a dissociation phase of 400 s. Shown are sensorgrams for binding of DBLβ3_D4 to ICAM-1_D1-D2 (**A**), ICAM-1_D1 (**B**), and 24E9 Fab (**C**). Data (black lines) are modeled to a one-site model (red lines). (**D**) Sensorgrams observed for the sequential binding of 24E9 Fab fragment and ICAM-1_D1 to immobilized PFD1235w DBLβ3_D4. Zero response is taken as the start of injection of ICAM-1_D1. A sensorgram for the binding of ICAM-1_D1 in the absence of 24E9 Fab is shown in red. (**E**) Quantification of the amount of ICAM-1_D1 binding to PFD1235w DBLβ3_D4 after preincubation of the DBLβ3_D4 with different concentrations of 24E9 Fab. (**F**) The ability of 24E9 mAb to inhibit DC4 DBLβ3_D4 domains binding to ICAM-1–Fc as assayed by ELISA. Mouse IgG was added as control. Mean OD values are shown for three independent experiments. Error bars indicate SD.

Binding to ICAM-1 is mediated through the DBLβ3_D4 domains of DC4 PfEMP1 (12). We therefore tested whether the 24E9 mAb blocks this interaction. We first compared the affinity of DBLβ3_D4 for both 24E9 and ICAM-1 by SPR. We used 24E9 Fab fragments, leading to monovalent binding, which allowed for global fitting of the data with a one-site binding model. 24E9 Fab bound to DBLβ3_D4 with low nanomolar affinity, comparable with the affinity of DBLβ3_D4 for ICAM-1_D1-D2 and ICAM-1_D1 (Fig. 3A, Table I). Furthermore, the interaction between 24E9 Fab and DBLβ3_D4 showed fast association and slow dissociation rates similar to those observed for the interaction between ICAM-1_D1 and DBLβ3_D4 (Fig. 3A–C, Table I).

**Table I.** Kinetic parameters derived from SPR experiments on ICAM-1_D1-D2, ICAM-1_D1, and 24E9 mAb interacting with PFD1235w DBLβ3_D4

Interaction	*k*_a_ (×10^5^ M^−1^ s^1^)	*k*_d_ (×10^−4^ s^−1^)	*K*_D_ (nM)	Model
ICAM-1_D1-D2::DBLβ3_D4	2.12	16.74	7.90	One-site
ICAM-1_D1::DBLβ3_D4	14.4	33.73	2.34	One-site
24E9 Fab::DBLβ3_D4	2.25	7.58	3.37	One-site

We next analyzed whether 24E9 mAb directly inhibits the DBLβ3_D4::ICAM-1 interaction. A chip coupled with DBLβ3_D4 was preincubated with different concentrations of 24E9 Fab, followed by injection of ICAM-1_D1. As a control, ICAM-1_D1 was flowed over the chip surface without prior incubation with 24E9 Fab (Fig. 3D, red curve). Preabsorption of DBLβ3_D4 with increasing concentrations of 24E9 Fab reduced the binding of ICAM-1_D1 in a concentration-dependent manner (Fig. 3D, 3E), demonstrating that 24E9 Fab effectively blocks the interaction between DBLβ3_D4 and ICAM-1_D1.

The observation that 24E9 is cross-reactive against several DC4 DBLβ3_D4 domains from different parasite isolates (Fig. 2) raised the possibility that 24E9 also cross-inhibits the interaction between ICAM-1 and these domains. We tested this by ELISA and found that 24E9 mAb inhibited ICAM-1 binding of PFD1235w DBLβ3_D4 and of the five DC4 DBLβ3_D4 domains in a concentration-dependent manner (Fig. 3F). Taken together, these data show that 24E9 mAb is both cross-reactive and cross-inhibitory of ICAM-1 binding to all tested DC4 DBLβ3_D4 domains and binds with a sufficiently strong affinity to effectively compete with ICAM-1 binding.

### 24E9 mAb and 24E9 Fab inhibits IE binding to ICAM-1 under flow conditions

**FIGURE 4.**
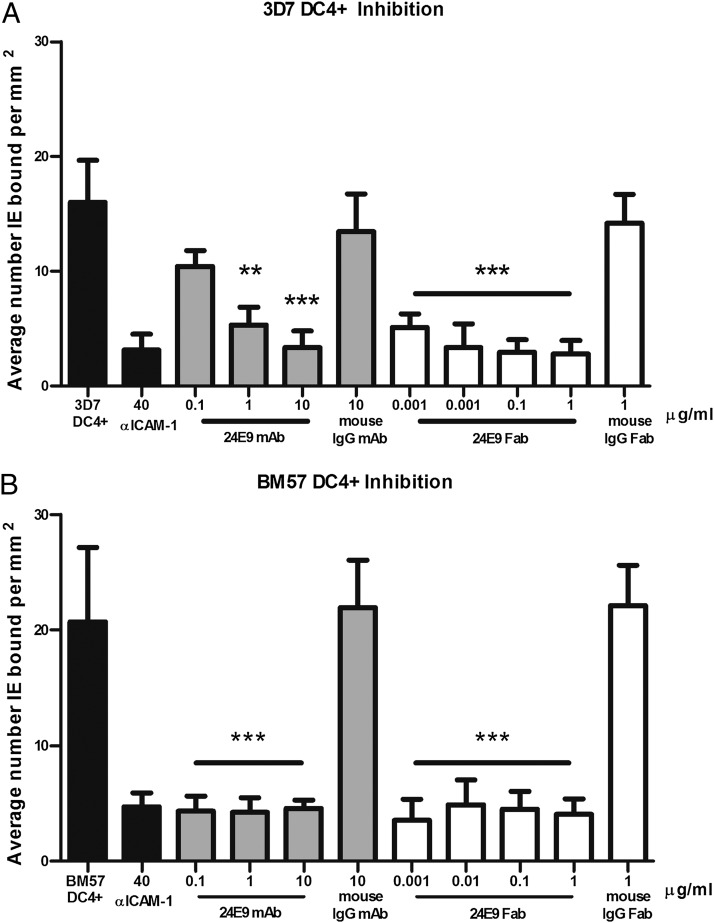
24E9 mAb and 24E9 Fab inhibit IE binding to ICAM-1 under flow conditions. Inhibition of adhesion by 24E9 mAb and 24E9 Fab of 3D7 DC4^+^ (**A**) and BM57 DC4^+^ (**B**) to rICAM-1 coated onto Biochips. Abs were titrated at 0.1–10 μg/ml (24E9 mAb) and 0.001–1 μg/ml (24E9 Fab). Each condition was run in triplicate for a minimum of three independent experiments and expressed as average number bound per square millimeter compared with untreated controls. Mouse IgG and mouse IgG Fab fragments were added as controls. Statistical significance was determined via one-way ANOVA with Tukey’s multiple comparison test. **p < 0.05, ***p = 0.0001.

IE expressing DC4 PfEMP1 proteins adhere to ICAM-1 (12), a phenotype linked to sequestration of IE in the microvasculature of the brain (6, 35). We therefore tested whether the 24E9 Ab blocks this interaction. Biochips were coated with recombinant ICAM-1, and 3D7 DC4^+^ parasites were flowed over at 1 dyn/cm^2^. The 24E9 mAb successfully inhibited adhesion at 1 μg/ml (67%; 133 nM) and at 10 μg/ml (79%; 1.3 mM), whereas the control mouse IgG (10 μg/ml) failed to significantly alter adhesion to ICAM-1 (Fig. 4A). Fab fragments generated from 24E9 mAb were also assessed for inhibition at 1 μg/ml (83%; 400 nM) and were titrated to determine the extent of activity. The Fab fragment continued to demonstrate adhesion inhibition at >50% even at 0.001 μg/ml (67%; 400 pM; Fig. 4A), whereas the control mouse IgG Fab again failed to alter adhesion at the highest concentration tested (1 μg/ml). A second strain, BM57 DC4^+^, was assessed, and like 3D7 DC4^+^, adhesion was significantly inhibited by 24E9 mAb (0.1 μg/ml; 80% inhibition) and 24E9 Fab (0.001 μg/ml; 83% inhibition) at the lowest concentrations tested (Fig. 4B). The specificity of adhesion to rICAM-1 was verified by preincubating control channels with anti–ICAM-1, which significantly reduced adhesion (81% inhibition; Fig. 4).

### 24E9 mAb recognizes a conformational epitope

**FIGURE 5.**
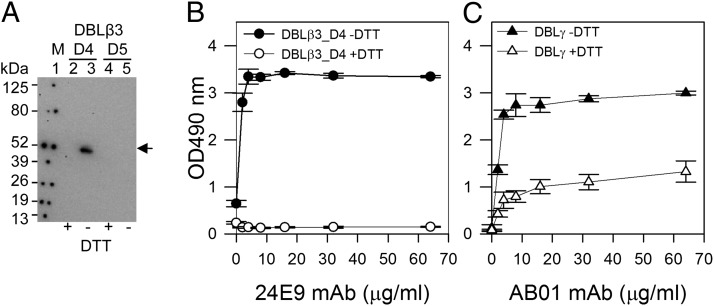
24E9 mAb recognizes a conformation epitope on PFD1235w DBLβ3_D4. (**A**) Western blotting of PFD1235w DBLβ3_D4 (D4) and DBLβ3_D5 (D5). +DTT (reduced), −DTT (nonreduced). *Lane 1*, Prosieve protein marker (M) visualized by phosphorescent paint as dots. *Lanes 2* and *3*, DBLβ_D4 (±DTT). *Lanes 4* and *5*, DBLβ3_D5 (±DTT). Arrow shows nonreduced DBLβ3_D4 (*lane* 3) recognized by 24E9 mAb. (**B**) 24E9 mAb ELISA reactivity against reduced (+DTT) and nonreduced (−DTT) PFD1235w DBLβ3_D4. (**C**) AB01 mAb ELISA reactivity against reduced (+DTT) and nonreduced (−DTT) DBLγ of PFD1235w. Mean OD values are shown for three independent experiments. Error bars indicate SD.

To determine whether 24E9 mAb interacts with a conformational epitope, we used Western blotting to test the reactivity of 24E9 mAb to reduced and nonreduced DBLβ3_D4. As a control, we performed the same experiment with the non-DC4 PFD1235w DBLβ3_D5 domain. 24E9 recognized only nonreduced DBLβ3_D4 (Fig. 5A). We observed the same result by ELISA (Fig. 5B), where 24E9 recognized only nonreduced DBLβ3_D4. To test whether the loss of reactivity of the mAb toward DTT-treated PFD1235W DBLβ3_D4 was a result of the mAb being reduced in the ELISA, we performed the same assay using the PFD1235w DBLγ-specific human mAb (AB01), which is only partially dependent on the correct folding of DBLγ (24). AB01 mAb was still able to recognize DTT-treated DBLγ (Fig. 5C) showing that a similar mAb remained intact in the ELISA. This suggests that 24E9 mAb targets a conformational epitope.

### The epitope targeted by 24E9 partially overlaps with the potential ICAM-1 binding site of PFD1235w DBLβ3_D4

**FIGURE 6.**
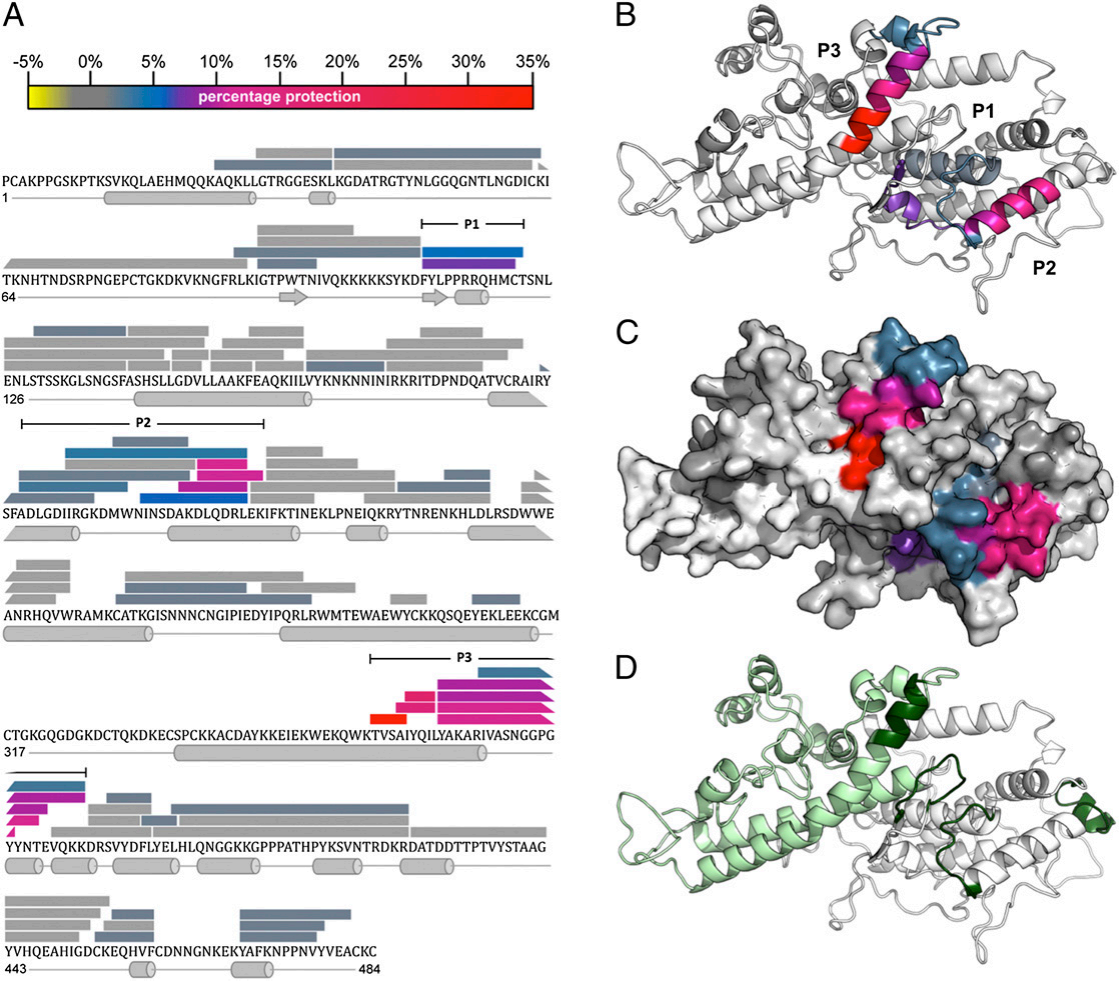
The 24E9 Fab binds to the convex surface of PFD1235w DBLβ3_D4. (**A**) Peptides from DBLβ3_D4 that were identified in mass spectra are represented by bars overlaying the primary sequence. The secondary structure, derived from a homology model of DBLβ3_D4, is shown below the sequence. The level of protection of individual peptides, as determined by comparing the %D incorporation over 200 min for free DBLβ3_D4 with that for DBLβ3_D4 bound to 24E9 Fab, is color coded according to the scale bar. Highly protected areas are in red, whereas unprotected areas are in gray. Three highly protected regions were P1 (residues 110–121), P2 (193–220), and P3 (357–388), as indicated. (**B**) HDX MX results were mapped onto a model of the PFD1235w DBLβ3_D4 domain. Protected areas are color coded as shown in (A). (**C**) Surface of PFD1235w DBLβ3_D4 model as shown in (B). (**D**) Potential ICAM-1 binding sites on the DBLβ3_D4 model as predicted by Bertonati and Tramontano (18) (green) and determined by Bengtsson et al. (12) (light green).

To identify the specific peptides and surface features recognized by 24E9, we used HDX MS, a powerful, modern immunological method to examine epitopes bound by Abs under native conditions (36, 37). We analyzed the DBLβ3_D4::24E9 Fab complex by measuring deuterium uptake over 200 min deuteration time for 83 partly overlapping peptides from DBLβ3_D4, alone or in complex with 24E9 Fab. These correspond to 79% of the DBLβ3_D4 primary sequence (Fig. 6A). Comparison of the level of deuteration highlighted three distinct regions, P1, P2, and P3, which show a reduction in deuterium uptake in the complex with 24E9 when compared with that of free DBLβ3_D4 (Fig. 6A), indicating that these regions are masked by 24E9 Fab. Similar results were also observed for 20 min deuteration time (data not shown). P1 is located in the N-terminal third of DBLβ3_D4 (subdomain 1), P2 in the center region (subdomain 2), whereas P3 is near the C terminus of the protein and part of subdomain 3. When mapped on a homology model of DBLβ3_D4, all three regions cluster to a well-defined, surface-exposed area (Fig. 6B, 6C), in accordance with the observation that 24E9 targets a conformational epitope (Fig. 5A–C). The size of this protected area is 2887 Å^2^, which is comparable with the total ∼2800 Å^2^ surface-exposed area of the variable loops of a Fab fragment.

The area protected by 24E9 Fab lies on the convex surface of DBLβ3_D4. Mutational and modeling studies of non-DC4 DBLβ domains previously showed that this surface contains the ICAM-1 binding site (18–20, 38). Amino acids equivalent to residues important for the interaction between group B DBLβ domains and ICAM-1 (Fig. 6D, dark green) (18) partly overlap with P1, P2, and P3. Furthermore, the ICAM-1 binding site of DBLβ3_D4 has been mapped to the C-terminal third of the domain (Fig. 6D, light green) (12). This includes region P3, which shows the strongest protection from deuteration in the DBLβ3_D4::24E9 Fab complex. These observations indicate that the epitopes targeted by 24E9 overlap with the ICAM-1 binding site of DBLβ3_D4.

**FIGURE 7.**
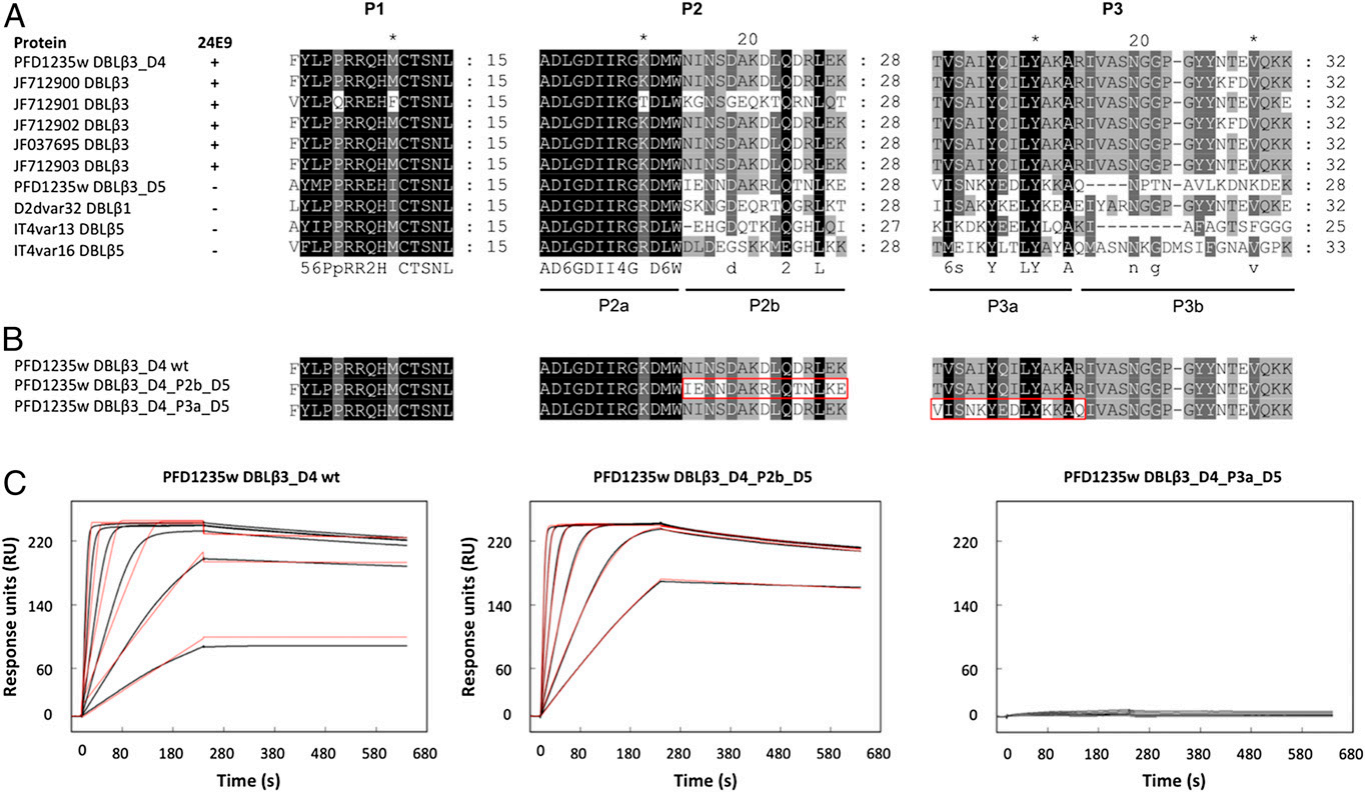
Fine mapping of the 24E9 binding site on DBLβ3_D4. (**A**) Sequence conservation of P1, P2, and P3 among DBLβ domains. Sequences were aligned with MUSCLE. The ability of the DBLβ domains to bind to 24E9 is indicated. (**B**) PFD1235w DBLβ3_D4 mutants were generated to determine the importance of protected peptides in the 24E9 mAb binding site. (**C**) SPR analysis of 24E9 mAb binding site mutants. 24E9 mAb was immobilized to 230 RU on a CM5 chip precoupled with protein G. Analytes were flowed over the chip surface at 40 μl/min with 240 s association and 400 s dissociation times. Data (black curves) were fitted to a one-site model (red curves).

To identify which of the protected peptides of DBLβ3_D4 makes the most significant contribution to the binding affinity, we first compared the P1, P2, and P3 regions between DBLβ3 domains that are recognized by 24E9 and those that are not (Fig. 7A). This revealed two motifs, P2b and P3a, which are mostly conserved only among 24E9 binding DBLβ3 domains (Fig. 7A) and are strongly protected in the DBLβ3_D4::24E9 Fab complex, suggesting that these motifs might directly contribute to 24E9 binding. To test this, we generated mutants by swapping the P2b and P3a peptides from DBLβ3_D4 for the equivalent regions of the DBLβ3_D5 domain (Fig. 7B), which is not recognized by 24E9. These mutants were expressed and purified as native DBLβ3_D4, and their folding was confirmed by CD spectroscopy (Supplemental Fig. 1). The binding of the mutants to 24E9 mAb was analyzed by SPR, which showed that the exchange of the P2b peptide had little effect on 24E9 affinity, whereas exchange of the P3a led to a complete loss of Ab binding (Fig. 7C, Table II). This demonstrates that P2 makes a minor contribution to 24E9 binding, whereas the P3 region contains an essential determinant of Ab binding.

**Table II.** Kinetic parameters derived from SPR experiments on 24E9 mAb interacting with mutant version of PFD1235w DBLβ3_D4

Interaction	*k*_a_ (×10^7^ M^−1^ s^−1^)	*k*_d_ (×10^−5^ s^−1^)	*K*_D_ (pM)	*R*_max_ (RU)	Model
DBLβ3_D4 wild type::24E9 mAb	6.4	7.7	1.2	229.2	One-site
DBLβ3_D4_P2b_D5::24E9 mAb	1.87	4.1	2.19	239.2	One-site
DBLβ3_D4_P3a_D5::24E9 mAb	2.2	24	110	8.64	One-site

### Low-resolution structure of the DBLβ3_D4::24E9 Fab complex

**FIGURE 8.**
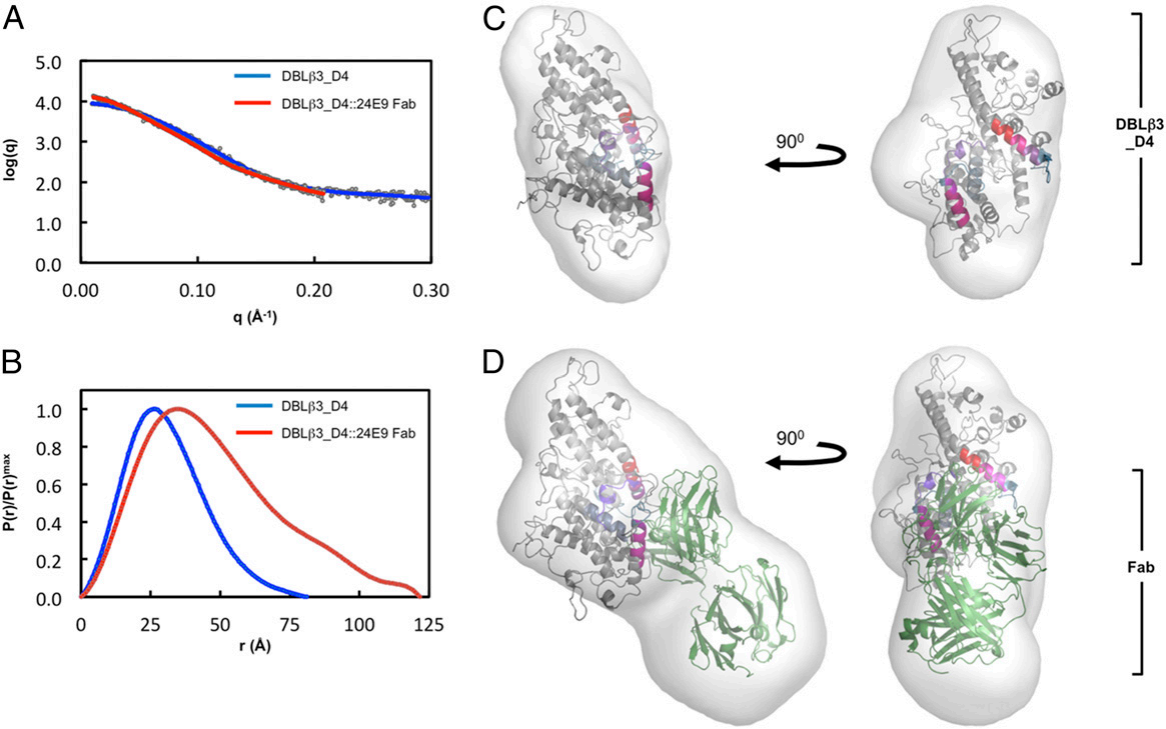
Molecular architecture of the PFD1235w DBLβ3_D4:24E9 Fab complex. (**A**) Theoretical scattering curves derived from ab initio models (solid lines) of DBLβ3_D4 and DBLβ3_D4::24E9 Fab superimposed on the experimental scattering data (circles). (**B**) Normalized distance distribution function P(r) for DBLβ3_D4 and DBLβ3_D4::24E9 Fab. (**C** and **D**) The homology model of DBLβ3_D4 was docked into ab initio envelopes of (C) DBLβ3_D4 alone and (D) the DBLβ3_D4::24E9 Fab complex. The peptides identified as being protected in the DBLβ3_D4::24E9 Fab complex are color coded as in Fig. 5. The structure of a mouse Fab fragment (green, PDB ID 3GK8) was used as a model for 24E9 Fab.

To understand better the architecture of the DBLβ3_D4::ICAM-1 complex, we performed small-angle x-ray scattering analysis of the DBLβ3_D4 domain alone or in complex with 24E9 Fab (Fig. 8). The Rg determined from the composite scattering curve (Fig. 8A) was higher for the DBLβ3_D4::24E9 Fab complex than for DBLβ3_D4 alone (Table III). The increased Porod volume and the apparent molecular mass were also consistent with formation of a 1:1 complex between 24E9 Fab and DBLβ3_D4 (Table III). The distance distribution function shows a more skewed profile for DBLβ3_D4::24E9 Fab than that for DBLβ3_D4 alone (Fig. 8B), indicating that binding of 24E9 Fab results in a more elongated particle (39). Accordingly, the *D*_max_ increases from 9.4 nm for DBLβ3_D4 to 12.2 nm for the complex (Table III).

**Table III.** Experimental values derived from PFD1235w DBLβ3_D4::24E9 Fab SAXS experiments

	Rg_exp_ (nm)	*D*_max_ (nm)	V_porod_ (nm^3^)	Mr_exp_ (kDa)	Mr_app_ (kDa)	χ
DBLβ3_D4	2.69	9.39	91.13	55	54.45	4.57
DBLβ3_D4::24E9 Fab	3.81	12.16	140.17	105	91.05	3.456

The experimental Rg (Rg_exp_) was determined using AutoRG (8), the *D*_max_ was derived from GNOM (30), and the Porod volume (V_porod_) was determined by using PRIMUS (29). The expected molecular mass (Mr_exp_) is shown for DBLβ3_D4 and the DBLβ3_D4::24E9 complex. The apparent molecular mass (Mr_app_) was calculated from the volume excluded in the final DAMMIN (33) model divided by 2. The χ value represents the best fit of 20 low-resolution shape reconstructions using ab initio modeling.

Envelopes for DBLβ3_D4 and DBLβ3_D4::24E9 Fab were generated by ab initio modeling based on the scattering data, and the homology model of DBLβ3_D4 was manually docked into these envelopes together with a model Fab fragment. Comparison of the two envelopes reveals that the DBLβ3_D4:24E9 Fab complex is more elongated, with an additional mass protruding from DBLβ3_D4, corresponding to 24E9 Fab (Fig. 8C, 8D). Simultaneous docking into this envelope positions the Ag binding loops of the Fab toward the regions identified as 24E9 binding site by HDX MS analysis (Figs. 6, 8D). This low-resolution shape reconstruction further supports the conclusion that 24E9 targets the convex surface of DBLβ3_D4 and overlaps with its potential ICAM-1 binding site.

## Discussion

The presence of inhibitory Abs that bind to the variable surface Ag PfEMP1 correlates with naturally acquired, protective immunity against PfEMP1-mediated IE adhesion during severe malaria (38, 40, 41). However, the epitopes targeted by such functional Abs and the mechanism by which they prevent IE adhesion are still unknown. In this study, we used immunological and biophysical methods to demonstrate that an mAb raised against a single DC4 DBLβ domain recognizes epitopes conserved between DC4 DBLβ domains and prevents ICAM-1 binding by both purified domains and IE, occluding the ICAM-1 binding site on the surface of a DBLβ domain.

Of biological relevance, the 24E9 mAb and Fab fragments inhibit 3D7 DC4^+^ IE at picomolar to subnanomolar concentrations under physiological flow conditions (Fig. 4). Titration of the Abs not only confirmed the specificity of 24E9, but also illustrated how effective the Ab remained at low concentrations. Despite having only one Ag binding site, the 24E9 Fab is a more potent inhibitor of ICAM-1 binding than 24E9 mAb (Fig. 4A). This lower efficacy of the full-length and thus bulkier IgG molecule might be explained by steric hindrance and partially restricted access to the binding site of the native PfEMP1 as compared with the smaller Fab fragment. 24E9 also successfully inhibited the ICAM-1 adhesion of erythrocytes infected by a genetically distinct parasite, BM57 DC4^+^. The mAb 24E9 is therefore able to inhibit ICAM-1 binding of DC4 DBLβ3_D4 domains from a number of different parasite isolates, indicating that these domains share a common antigenic epitope.

Using HDX MS, we have identified three regions that cluster on the convex surface of PFD1235W DBLβ3_D4 and that show reduced hydrogen-deuterium exchange in the presence of 24E9. One of these three peptides, the P3a motif of region P3, was protected most strongly (Fig. 6) and is absolutely required for Ab binding (Fig. 7C). Indeed, this motif is strictly conserved only among DBLβ3 domains recognized by 24E9. In addition, the P2b motif of region P2 is conserved in most 24E9-binding DBLβ3 domains, but varies significantly between nonbinders (Fig. 7A). However, this motif plays only a minor role in 24E9 binding, as demonstrated by a slight reduction in *K*_D_ when it is mutated (Table II). In contrast, P1 and the remaining regions of P2 and P3 show a substantial degree of conservation between both 24E9-binding and nonbinding DBLβ domains (Fig. 7A), making it more likely that these amino acids are not part of the 24E9-binding site, but instead are sterically protected from hydrogen-deuterium exchange by the presence of the Ab binding to the neighboring epitopes. Our mapping data also suggest a mechanism by which 24E9 inhibits ICAM-1 binding, because epitopes recognized by mAb 24E9 cluster on the convex surface of DBLβ3_D4 that is predicted to contain the binding site for ICAM-1 (15, 19, 20, 38). In addition, region P3, which contains the main determinant of 24E9 binding, lies within subdomain 3 of DBLβ3_D4. This subdomain forms a significant part of the convex surface of DBLβ3_D4, and a previous study suggested that it is required for the interaction with ICAM-1 (12). These findings indicate that 24E9 exerts its inhibitory function by masking the ICAM-1 binding site of DBLβ3_D4. These conclusions are further supported by our low-resolution shape reconstruction, determined by SAXS, which shows that 24E9 Fab adopts an orientation relative to the DBLβ3_D4 that is similar to that of ICAM-1_D1-D2 bound to the DBLβ domain of IT4var13 (19).

The identification of the convex surface of DBLβ domains as the main target of inhibitory Abs provides important knowledge for choosing the components of a vaccine aimed at preventing PfEMP1-mediated adhesion of IE during severe malaria. A detailed mapping and structural characterization of the ICAM-1 binding sites of DBLβ domains from group A and B PfEMP1 and the identification of conserved surface features involved in this interaction are now needed to guide future decisions about how to design immunogens that elicit Abs inhibitory of ICAM-1 binding. Our observation that an Ab raised against a single DBLβ3_D4 domain prevents the interaction between ICAM-1 and DBLβ3_D4 domains from genetically distant parasite isolates demonstrates the existence of conserved antigenic epitopes. These might be used to specifically induce the production of Abs that cross-inhibit ICAM-1 binding by an important set of ICAM-1 binding DBLβ domains. Because DC4 DBLβ3_D4 domains are found in group A PfEMP1, which have been associated with increased IE adhesion and severe malaria (6, 15, 42), such conserved epitopes are promising candidates for inclusion in a vaccine that interferes with the PfEMP1::ICAM-1 interaction and confers strain-independent protection against severe malaria.

## Supplementary Material

Data Supplement
