# Supplementary material for: Mapping the Binding Site of a Cross-Reactive Plasmodium falciparum PfEMP1 Monoclonal Antibody Inhibitory of ICAM-1 Binding
Source: J Immunol. 2015 Aug 28;195(7):3273–83. doi: 10.4049/jimmunol.1501404 (PMC4574524; doi:10.4049/jimmunol.1501404)
Supplement: Data Supplement [file JI_1501404.zip › JI_1501404_Supplemental_Figure_1.pdf]

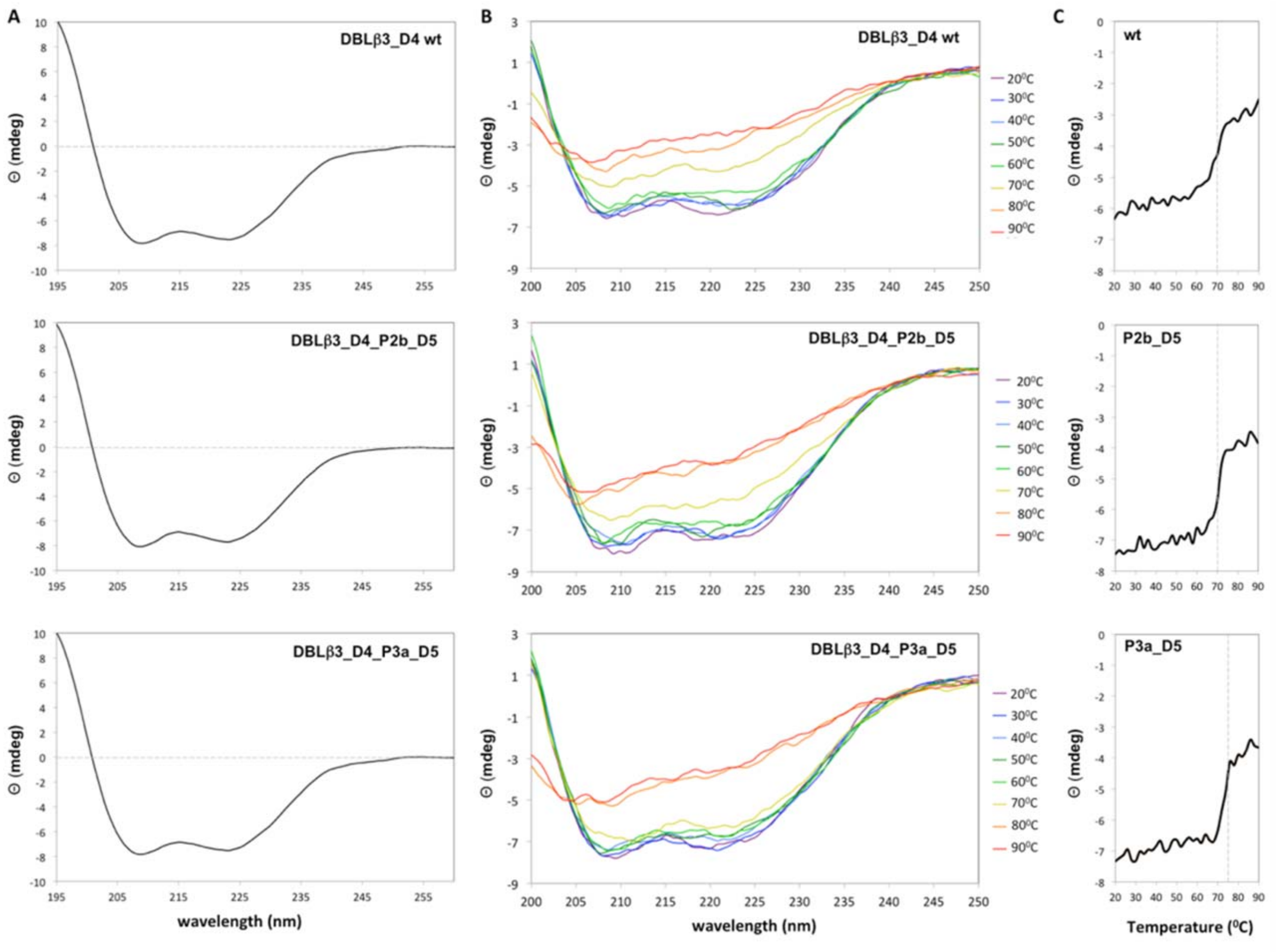

Fig. S1. CD analysis of DBLβ3_D4 wt, DBLβ3_D4_P2b_D5 and DBLβ3_D4_P3a_D5.

(A) Secondary structure analysis of DBLβ3_D4 wt, DBLβ3_D4_P2b_D5 and DBLβ3_D4_P3a_D5. Circular dichorism spectra were recorded between 195 and 260 nm at 20°C. For each sample, four measurements were averaged and corrected for buffer absorption. **(B)** Thermal denaturation of DBLβ3_D4 wt, DBLβ3_D4_P2b_D5 and DBLβ3_D4_P3a_D5. Spectra were recorded between 200 and 250 nm. Between each measurement, the temperature was increased in 0.5°C increments. **(C)** Melting curve for DBLβ3_D4 wt, DBLβ3_D4_P2b_D5 and DBLβ3_D4_P3a_D5. The ellipticity θ was measured at 222 nm from 20°C to 90°C. Between each measurement, the temperature was increased in 0.5°C increments.
